# Development of a PHBV nanoparticle as a peptide vehicle for NOD1 activation

**DOI:** 10.1080/10717544.2021.1923862

**Published:** 2021-06-01

**Authors:** Mauricio Cabaña-Brunod, Pablo A. Herrera, Valeria Márquez-Miranda, Felipe M. Llancalahuen, Yorley Duarte, Danilo González-Nilo, Juan A. Fuentes, Cristián Vilos, Luis Velásquez, Carolina Otero

**Affiliations:** a Escuela de Química y Farmacia, Facultad de Medicina, Universidad Andres Bello, Santiago, Chile; b Center for Applied Nanotechnology, Faculty of Sciences, Universidad Mayor, Huechuraba, Santiago, Chile; c Center for Bioinformatics and Integrative Biology, Facultad de Ciencias de la Vida, Universidad Andrés Bello, Santiago, Chile; d Laboratorio de Fisiopatología Integrativa, Facultad de Ciencias de la Vida, Universidad Andres Bello, Santiago, Chile; e Laboratorio de Genética y Patogénesis Bacteriana, Departamento de Ciencias Biológicas, Facultad de Ciencias de la Vida, Universidad Andrés Bello, Santiago, Chile; f Laboratory of Nanomedicine and Targeted Delivery, School of Medicine, Universidad de Talca, Talca, Chile; g Center for the Development of Nanoscience and Nanotechnology (CEDENNA), Universidad de Santiago de Chile, Santiago, Chile; h Instituto de Investigación Interdisciplinar en Ciencias Biomédicas SEK, Facultad de Ciencias de la Salud, Universidad SEK, Santiago, Chile

**Keywords:** Nanoparticles, macrophage, innate immunity, Nod1 agonist, PHBV

## Abstract

NOD1 is an intracellular receptor that, when activated, induces gene expression of pro-inflammatory factors promoting macrophages and neutrophils recruitment at the infection site. However, iE-DAP, the dipeptide agonist that promotes this receptor's activation, cannot permeate cell membranes. To develop a nanocarrier capable of achieving a high and prolonged activation over time, iE-DAP was encapsulated in nanoparticles (NPs) made of poly (3-hydroxybutyrate-co-3-hydroxyvalerate) (PHBV). The physicochemical properties, colloidal stability, encapsulation efficiency, and cellular uptake of iE-DAP-loaded PHVB NPs were analyzed. Results evidenced that physicochemical properties of iE-DAP-loaded NPs remained stable over time, and NPs were efficiently internalized into cells, a process that depends on time and concentration. Moreover, our results showed that NPs elicited a controlled cargo release *in vitro*, and the encapsulated agonist response was higher than its free form, suggesting the possibility of activating intracellular receptors triggering an immune response through the release of NOD1 agonist.

## 1. Introduction

Innate immunity is the first line of defense in a host that promotes resistance against pathogens. It consists of the immediate response of constant and non-specific intensity that requires different types of mechanisms and elements in action, which prevents the entry of pathogens into the body and their proliferation. Among those elements are external chemical, physical, and biological barriers (skin, mucous membranes, body secretion enzymes such as lysozyme, lactoperoxidase, intestinal, vaginal microbiota, and pH) (O'Hara & Shanahan, 2006). When this first defensive barrier is overcome, the activation of internal innate mechanisms (soluble factors and cellular components) is triggered, avoiding the pathogen's establishment, development, and action. In that case, immune cells such as monocytes, neutrophils, and macrophages are responsible for phagocytizing the pathogen at the infection site (Stossel, 1974). Moreover, those immune cells release cytokines (TNF-α, IL-1β, IL-6, IL-12) to generate an inflammatory response, while other humoral components, such as the complement, antimicrobial peptides (defensins), and the acute phase proteins participate in the process of recognition and elimination of the pathogen.

Pathogen recognition receptors (PRR) are responsible for detecting different microorganisms by recognizing conserved structures, known as pathogen-associated molecular patterns (PAMPs). PAMPs, which initiate an innate immune response during infection (Motta et al., 2015), compromise members of the nucleotide-linked oligomerization domain type receptors (NLR) family. NLR family consists of 22 cytoplasmic pathogen sensors, which are characterized by having a trimeric structure. NOD1 and NOD2 receptors belong to an NLR subfamily, both containing LRR and NOD domains, and differ only in one or two copies of the caspase activation recruitment domains (CARDs). Both NOD1 and NOD2 exert a fundamental role in the defense against bacterial infections and regulate the host inflammatory response (Charlotte et al., 2013). NOD2 agonists are highly pyrogenic; thereby, their use in humans has been restricted (Monie, 2017). Since it is not the case for NOD1 agonists, these represent an attractive focus to obtain a more controlled response.

Inohara et al. (2001) proposed that NOD1 and NOD2 receptors are intracellular sensors of bacterial lipopolysaccharide (LPS). However, in subsequent studies, they concluded that NOD receptors detect the peptidoglycan fragments from the bacterial cell wall (Chamaillard et al., 2003; Inohara et al., 2003). One of the NOD1 agonists is the dipeptide γ-d-glutamyl-*meso*-diaminopimelic acid (iE-DAP), which is found in the peptidoglycan of Gram-negative bacilli and particular Gram-positive bacteria such as *Bacillus subtilis* and *Listeria monocytogenes*. iE-DAP has been shown to stimulate the immune system by triggering a signaling cascade that leads to NF-κB activation and inflammatory cytokines production (Chamaillard et al., 2003; Girardin et al., 2003).

The innate immune system's action begins with the activation of NOD1 by the binding of its bacterial ligand (iE-DAP) to the LRR domain. This event initiates a process of auto-oligomerization in its central zone, then interacts with its protein-2 adapter molecule (RIP2, receptor-interacting protein 2), a serine/threonine kinase that binds to the CARD domain of the NOD1 receptor (Girardin et al., 2001; Kobayashi et al., 2002; Hasegawa et al., 2008). Recruitment of RIP2 at this site of interaction is essential, as it communicates the signaling cascade's initiation through NF-κB or via the mitogen-activated protein kinase (MAPK) (Ogura et al., 2001; Kobayashi et al., 2002).

The use of an effective transport and release system of adjuvants, such as NPs based on biodegradable and biocompatible polymers, would increase the efficiency of poorly soluble or highly toxic compounds, reducing, in turn, their side effects in addition to protecting the compound through encapsulation (Kumari et al., 2010). There are a variety of polymers that can be used; the best known is poly(lactic-*co*-glycolic acid) (PLGA) and poly(lactic acid) (PLA). These polymers have been approved for humans by the Food and Drug Administration (FDA) (Panyam & Labhasetwar, 2003). Remarkably, the poly(3-hydroxybutyrate-*co*-3-hydroxyvalerate) (PHBV) has been shown to generate a prolonged and controlled release of the encapsulated compounds (Vilos & Velasquez, 2012). PHBV has been extensively studied as a biomaterial in microparticle-based drug transport systems (Sendil et al., 1999; Vilos & Velasquez, 2012; Vilos et al., 2012). It has the advantage of being biodegradable, non-toxic, and with a low production cost compared to the other polymers such as PLGA (Slater et al., 1999). It has been observed that PHBV nanoparticles allow controlled release of the encapsulated content at short times, being a strategic option to be used as modulators in the activation of the innate immune system *in vitro* (Peñaloza et al., 2017). Efficient internalization of these nanoparticles would be under endocytic mechanisms, where their incorporation, traffic, destination, and cell degradation would be dependent on physicochemical properties of NPs (Sahay et al., 2010; Yameen et al., 2014). Due to the fact that the iE-DAP agonist is not permeable to the membrane, we propose that PHBV nanoparticles might deliver the iE-DAP agonist into the cell cytoplasm in a sustained manner over time, allowing the activation of the NOD1 receptor reflected by the secretion of pro-inflammatory cytokines.

## 2. Materials and methods

### 2.1. Materials

Poly(3-hydroxybutyric acid-*co*-hydroxyvaleric acid) (PHBV) 12% w/w poly-3-hydroxyvalerate (PHV); polyvinyl alcohol (PVA) (average mol wt. 30,000–70,000); *Escherichia coli O55* lipopolysaccharide: B5; TWEEN 20 and 0.45 µm Millipore Filters were purchased from Sigma-Aldrich (St. Louis, MO). Trypan Blue stain 0.4%; antibiotic–antifungal (100×); bovine fetal serum; trypsin-EDTA 1× and Nile Red 552/636 were purchased from Gibco by Life Technologies (Carlsbad, CA). Hoechst 33342 was purchased from Invitrogen (Carlsbad, CA). EDTA was purchased from Calbiochem (San Diego, CA). PBS was purchased from Winkler (Taipei City, Taiwan). Dichloromethane, sodium bicarbonate, sulfuric acid, hydrogen peroxide, Triton X-100, and Amicon Ultra centrifugation filters were purchased from Merck (v). CytoTox 96 Non-Radioactive LDH Cytotoxicity Assay was purchased from Promega (Madison, WI). IL-6 Human ELISA MAX Deluxe Set, Biolegend (San Diego, CA); TNF-α Human ELISA MAX Deluxe Set, Biolegend, and Nunc MaxiSorp ELISA Plate were purchased from Biolegend (San Diego, CA). NOD1 Agonist γ-d-Glu-mDAP (iE-DAP) were purchased from Invivogen (San Diego, CA).

### 2.2. Preparation of iE-DAP–loaded PHBV NPs

PHBV NPs were developed using a water-oil-water double emulsion method (Vilos et al., 2012). Briefly, in one vial, 1 ml of PHBV (3 mg/ml) with 399 µl of MiliQ water + 1 µl of the iE-DAP ligand (5000 µg/ml) or 100 µl of RN (Red Nile) fluorophore (1000 µg/ml) was added. The first emulsion (w1/o1) was prepared by sonication (Sonic Vibra Cell, Equilab) for 40 s. The water-in-oil emulsion was further emulsified by sonication for 30 s under the same conditions in 2 ml of an aqueous solution of 5 mg/ml PVA (w2). The mix was deposited in 100 ml precipitated glass by adding 10 ml of water and subjected to orbital agitation at 300 rpm overnight (Multistirrer Magnetic Stirrer, Arquimed, Santiago, Chile). Solvent evaporation was performed at room temperature for 12 h. Formulated NPs were washed three times with 50 ml of MilliQ water using Amicon Ultra-4 centrifuge filters with a molecular weight of 100 kDa and then centrifuged again at 3000 rpm for 15 min, then particles were suspended in 1000 µl of MilliQ water and stored at 4 °C for fresh use or at −20 °C for a subsequent lyophilization under vacuum at −80° C for 8 h (Coimbra et al., 2008; Vilos et al., 2012).

### 2.3. PHBV NPs characterization by dynamic light scattering (DLS)

Each batch of formulated NPs was suspended in 1 ml of PBS 1× pH 7.4, and nanoparticle size (nm), polydispersity coefficient (PDI), and zeta potential (mV) were determined by the light scattering technique using a Nano-ZS Zetasizer (Malvern Instruments Ltd., Malvern, UK).

### 2.4. Encapsulation efficiency of iE-DAP in PHBV

Aliquots of 1 ml were taken from the supernatants of each batch. This volume was filtered into a vial to be determined by the UPLC Acquity system (Waters, Milford, MA) free iE-DAP agonist concentration. In parallel as a complementary methodology, 6 mg of PHBV NPs encapsulating iE-DAP were weighed and dissolved in a mixture of 10% (v/v) DCM/MeOH, and it was sonicated at an intensity of 30% for 5 min to degrade the NPs and release the encapsulated content. Subsequently, the suspension was centrifuged at 9000 rpm for 10 min, the supernatant was filtered and collected in a vial to quantify by UPLC the amount of iE-DAP ligand encapsulates in NPs of PHBV.

The encapsulation efficiency (% EE) was calculated as follows:

(1) $$\%EE=\frac{\mathrm{Real}iE-\mathrm{DAP}\mathrm{mass}\mathrm{obtained}by\mathrm{UPLC}}{10\mu g\mathrm{initial}\mathrm{concentration}ofiE-\mathrm{DAP}}\times 100$$

*Real iE-DAP mass obtained by UPLC: mass of the dipeptide iE-DAP encapsulated in the NPs-PHBV and quantified by UPLC as described.

*10 μg initial concentration of iE-DAP: mass of the initial iE-DAP dipeptide to encapsulate in the synthesis of NPs-PHBV.

### 2.5. Transmission electron microscopy (TEM)

NPs structure was also characterized using transmission electron microscopy. One drop of the NP sample was placed onto an ultra-thin Lacey carbon-coated 400-mesh copper grid and allowed to dry at room temperature for 10 min prior to image acquisition, ensuring no more than 1 min of electron beam exposure to the sample. TEM images were acquired using an LVEM5 electron microscope (Delong Instrument, Montreal, Quebec, Canada) at a nominal operating voltage of 5 kV. The small volume of the vacuum chamber in the LVEM5 microscope facilitates rapid sample visualization within 3 min before observation. The low voltage used delivers high contrast in soft materials (up to 20-fold) compared with high-voltage electron microscopes, which use accelerating voltages of approximately 100 kV; this procedure facilitates the emission of staining procedures and allows the direct visualization of biological samples. Digital images were captured using a Retiga 4000 R camera (QImaging, Inc., Tucson, AZ) at its maximal resolution.

### 2.6. Release profile of the iE-DAP over time

The *in vitro* kinetics release study of iE-DAP since PHVB NPs, 6 mg of iE-DAP–loaded PHBV NPs were suspended into 1 ml of PBS 1×, then incubated at 37 °C under constant agitation. At the times of 0, 1, 2, 3, 4, 6, 8, 10, and 12 h, it was centrifuged at 9000 rpm for 5 min, and 50 µl aliquots were removed, filtered, collected in a vial, and subsequently quantified by UPLC to determine the amount of iE-DAP agonist released over time. As a control, empty NPs were used.

### 2.7. Stability of NPs over time

Different batches of synthesized NPs were stored for 4 weeks at 4 °C, then at different times of 0, 1, 2, 3, and 4 weeks their physicochemical properties (size, potential Z, and PDI) were evaluated by DLS.

### 2.8. Cell culture

Raw 264.7 cell line (murine macrophages, ATCC TIB-71™) was grown in TR6002 bottles (Trueline, Hebron, IL) by adding 15 ml of RPMI-1640 medium supplemented with 10% v/v fetal serum bovine (FBS), 1 mM sodium pyruvate, and 1% v/v penicillin–streptomycin–amphotericin B. Cells were incubated at 37 °C and 5% CO_2_, changing the culture medium every 2–3 days and propagated when they reached between 80 and 90% confluence.

### 2.9. PHBV internalization assay

To quantify the internalization rate of NPs by cells, dye-loaded PHBV NPs were formulated by using the same conditions of iE-DAP-loaded PHBV NPs.

Nile Red is an uncharged hydrophobic molecule that functions as a fluorescent probe for intracellular lipids and hydrophobic proteins' domains. During the nanoparticles formulation process, Nile Red was dissolved together with the polymeric solution.

To avoid counting nanoparticles that could have remained attached to the cell surface, we did at least four washes using PBS prior to the quantification using cytometry. We have used this protocol successfully in previous articles (Peñaloza et al., 2017) and following recommendations from other sources (Fernando et al., 2010; Snipstad et al., 2017).

In the NPs uptake experiments, cells were incubated with 10, 50, 100, 500, and 1000 µg/ml of dye-loaded NPs, and analyzed at 1, 2, 4, 6, 24, and 48 h. Then, the supernatant was discarded, and cells were washed three times with PBS. Finally, cells were resuspended in 500 µl of PBS, and samples were analyzed by flow cytometry (BD Accuri™ C6, BD Biosciences, Franklin Lakes, NJ). Ten thousand events were determined using the FL2 detection filter.

### 2.10. LDH cytotoxicity assay

Empty PHBV NPs were weighed and reconstituted with culture medium (1 mg/ml). Then, they were sonicated for 30 s with an amplitude of 30% for its homogenization. Afterward, cells were incubated with 10, 50, 100, 500, and 1000 µg/ml of NPs for 24 or 48 h at 37 °C and 5% CO_2_. 45 min before completing the final incubation time, 100 µl of 10× lysis buffer was added to the positive control, and supernatants from cells were taken and centrifuged at 14,000 rpm for 5 min. About 50 µl aliquots of centrifuged and supernatants were transferred and mixed with 50 µl of mixed substrate for 30 min at room temperature. Then, 50 µl of stop buffer was added to stop the enzymatic reaction, and the absorbance at 490 nm was read in a spectrophotometry reader (Synergy H1 Hybrid Reader, Biotek®, Winooski, VT).

Absorbances were used to calculate the percentage of cytotoxicity of each sample by the following equation:

(2) $$\%\mathrm{cytotoxicity}=\frac{\mathrm{experimental}-\mathrm{negative}\mathrm{control}}{\mathrm{positive}\mathrm{control}-\mathrm{negative}\mathrm{control}}\times 100$$

experimental: absorbance of the experimental sample; *negative control: culture medium absorbance; *positive control: absorbance with lysis buffer.

### 2.11. Cellular viability by annexin V assay

Detection of phosphatidylserine translocated outside the cell membrane is a critical step in apoptosis. Phosphatidylserine can be labeled by fluorescein-annexin isothiocyanate V allowing the detection of dead cells by apoptosis (Moretti et al., 2014). To determine cell viability, cells were incubated with different concentrations (10, 50, 100, 500, and 1000 µg/ml) of empty PHBV NPs at different times (1, 2, 4, 6, 24, and 48 h). Phosphatidylserine residues translocated outside the cell membrane were detected by Annexin V conjugated with APC (BD Pharmingen™, San Diego, CA). Then, cells (5 × 10^5^) were washed with annexin V binding buffer (10 mM HEPES, pH 7.4; 280 nM NaCl; 5 mM CaCl_2_) and resuspended in the binding buffer; then Annexin V conjugated to APC (1 µg/ml) was added, mixed and allowed to incubate at 37 °C for 15 m. Samples were then analyzed by flow cytometry, and 10,000 events were evaluated using the FL4 detection filter (BD Accuri™ C6, BD Biosciences, San Diego, CA).

### 2.12. Immunofluorescence assay

The translocation of p65 subunit of cytoplasmic NF-κB into the nucleus was evaluated by fluorescence microscopy. Raw 264.7 cells were grown in coverslips in 24-well culture plates at a concentration of 5 × 10^5^ cells per well with different controls and treatments for 24 at 37 °C and 5% CO_2_. After that, cells were washed with PBS and fixed with 4% paraformaldehyde at room temperature for 10 min and then cells were permeabilized with 0.2% Triton X-100 for 10 min, prior to being washed twice with 0.2% PBS-BSA. About 1 µg/ml of rabbit polyclonal antibody was incubated against p65 subunit for 1 h at room temperature, then incubated the secondary antibody IgG conjugated with Alexa-fluor 488. Cells were also incubated with Hoechst 33342® (1 µg/ml) as a nuclear marker for 1 h at room temperature. Finally, 1 µl of Fluoromount-G™ was added to mount samples on coverslips. Samples were then observed by fluorescence microscopy (LSM 510, Carl Zeiss, Jena, Germany), and pictures were analyzed by Dimension cellSens v1.7.1 software (Olympus Corp., Tokyo, Japan). The blue to red color of the nucleus was exchanged through an image post-editing to visualize a better contrast with merge. To analyze the levels of nuclear translocation of the p65 subunit, immunofluorescence images were quantified through the Fiji ImageJ software (Wessel & Hanson, 2015).

### 2.13. ELISA

Experimental cultures carried out as the immunofluorescence assays, supernatants were collected and stored at −80 °C. Subsequently, the concentration of pro-inflammatory cytokines secreted into these supernatants was evaluated. ELISA assay was used to detect the protein expression of IL-6 and TNF-α following the manufacturer's instructions with mouse IL-6 and TNF-α ELISA kits (MAX Deluxe Set, Biolegend, San Diego, CA). The reaction was stopped by adding 100 µl of stop buffer (NH_2_SO_4_) (yellow coloring). The colorimetric reaction was read at 450 nm in a spectrophotometry reader (Synergy H1 Hybrid Reader, Biotek®, Winooski, VT).

### 2.14. Statistical analysis

All data were analyzed using GraphPad Prism version 5.03 software (GraphPad Software, La Jolla, CA). Results were analyzed using one-way ANOVA with subsequent Bonferroni test. ns = not significant, *p < .05, **p < .01, ***p < .001, ****p < .0001.

## 3. Results

### 3.1. Generation and characterization of iE-DAP-loaded PHBV NPs

**Figure 1.**
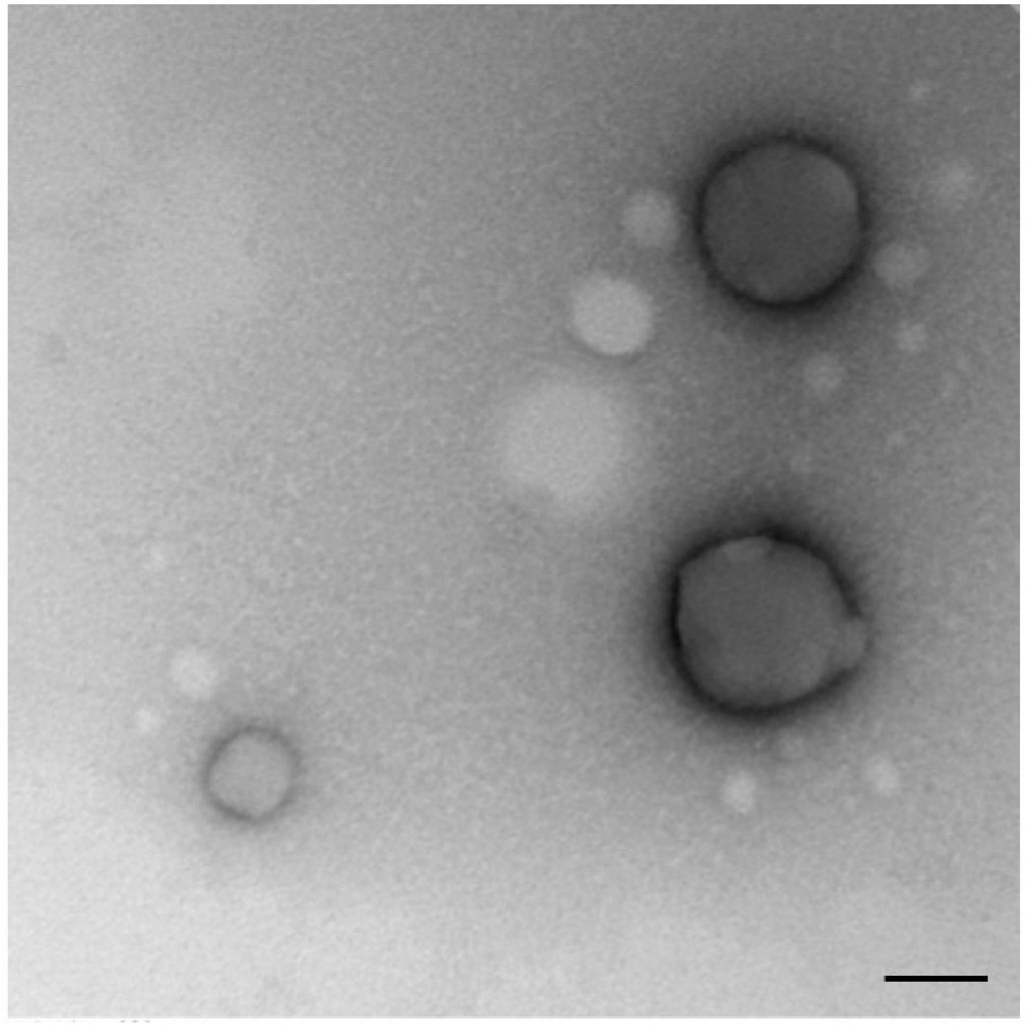
PHBV representative image of PHBV nanoparticles by transmission electron microscopy (TEM). Objective ×49,000 magnification. Bar size 100 nm.

NPs loaded with iE-DAP agonist formulation were obtained by the double emulsion method. To this end, an aqueous solution of iE-DAP was emulsified in PHBV previously dissolved in dichloromethane (DCM). A second emulsification step was performed by adding PVA as a surfactant, followed by sonication. The resulting solution was stirred to allow the evaporation of DCM. iE-DAP ligand encapsulation did not exert significant changes in the physicochemical properties of the NPs compared to the void ones (Table 1). Instead, low PDI values ​​indicated that the particle size distribution was homogeneous between 198 and 215 nm and had a negative Z potential of −6.5 to −12.5 mV, as shown in Table 1. With minimal differences in size, PDI, and Z potential among all synthesized NP batches, double emulsion protocol was reproducible. Shape and morphology were also analyzed by transmission electronic microscopy (Figure 1).

**Table 1.** Characterization of Functionalized PHBV NPs.

Formulation	Average size (nm)	PdI	Z Potential (mV)	Agonist encapsulation efficiency (%)	Agonist (µg)/ PHBV (mg)
PHBV (*n* = 10)	197.8 ± 3.15	0.12 ± 0.03	−12.5 ± 2.29	–	–
PHBV-RN (*n* = 10)	214.4 ± 4.97	0,28 ± 0.06	−6.5 ± 2.6	–	–
PHBV-iE-DAP (*n* = 10)	207.9 ± 5.1	0.13 ± 0.02	−8.01 ± 1.67	70.6 ± 7.2 (*n* = 3)	7.6 ± 0.7 (*n* = 3)

Size, size distribution (PdI), Z potential and nanoparticle load levels of NPs generated by nanoprecipitation are represented as the average ± standard deviation of several generated lots.

### 3.2. Assessment of the encapsulation efficiency of iE-DAP peptides in PHBV NPs

Two methods were performed to calculate the amount of iE-DAP agonists encapsulated in NPs. The first method consisted of UPLC quantification of free agonist molecules that were not encapsulated during the synthesis, remaining in the supernatant. The second method consisted of degrading PHBV-iE-DAP NPs by using organic solvents (DCM/MeOH), which allows the release of agonist molecules to be quantified. The encapsulation efficiency was 70.6 ± 7.2%, and the amount of iE-DAP ligand mass per nanoparticle weight was equivalent to 7.6 ± 0.7 µg/mg of PHBV, respectively (Table 1).

### 3.3. Stability of iE-DAP-loaded PHBV-NPs

**Figure 2.**
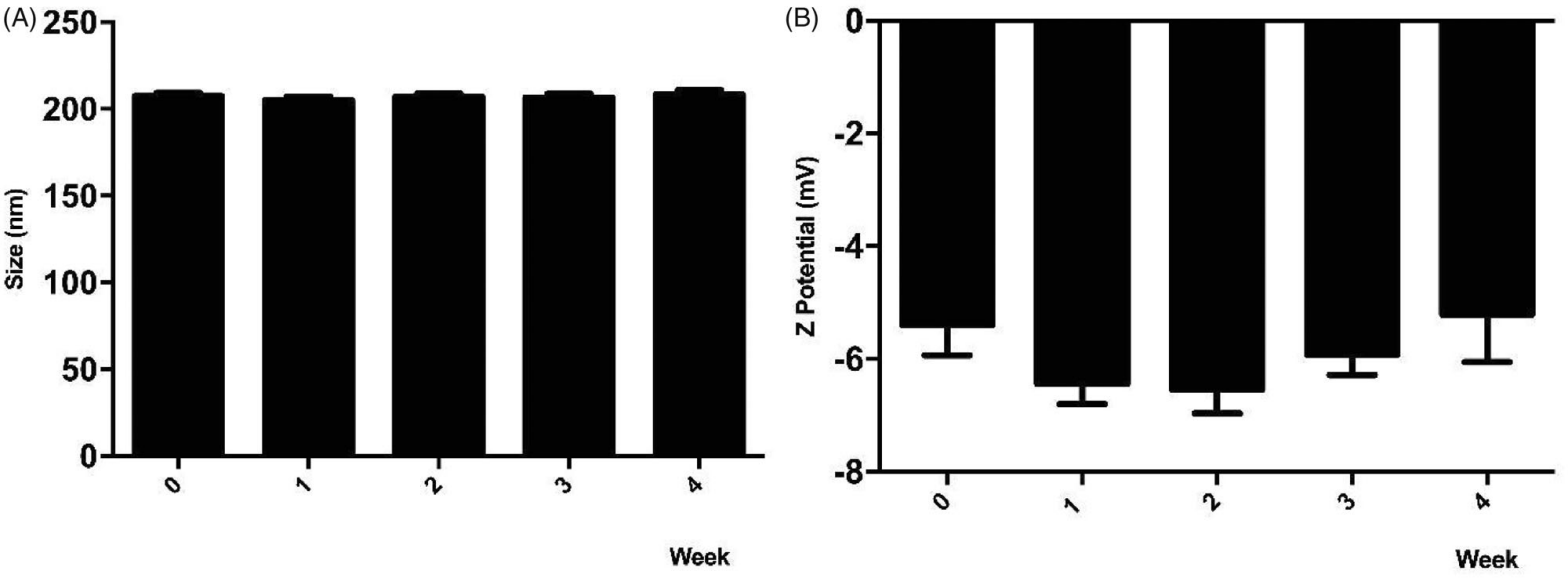
Stability of PHBV-iE-DAP NPs. The size and Z potential of different synthesized nanoparticles' batches were determined and stored at 4 °C for 4 weeks using DLS. Results are expressed as the mean ± standard deviation of triplicate determinations of three independent experiments. One-way ANOVA was performed with the Bonferroni test as statistical analysis. n.s. = not significant.

To preserve NPs under storage conditions for subsequent tests, the colloidal stability of the iE-DAP-loaded PHBV-NPs in PBS solution was determined for 4 weeks at 4 °C. Degradation of synthesized NPs can be observed both by changes in their size and surface charge distribution. As shown in Figure 2(A,B), results indicate no variations in the size and surface charge of the NPs in 4 weeks of storage, meaning that the compound is stable over time.

### 3.4. Release profile of the iE-DAP over time

**Figure 3.**
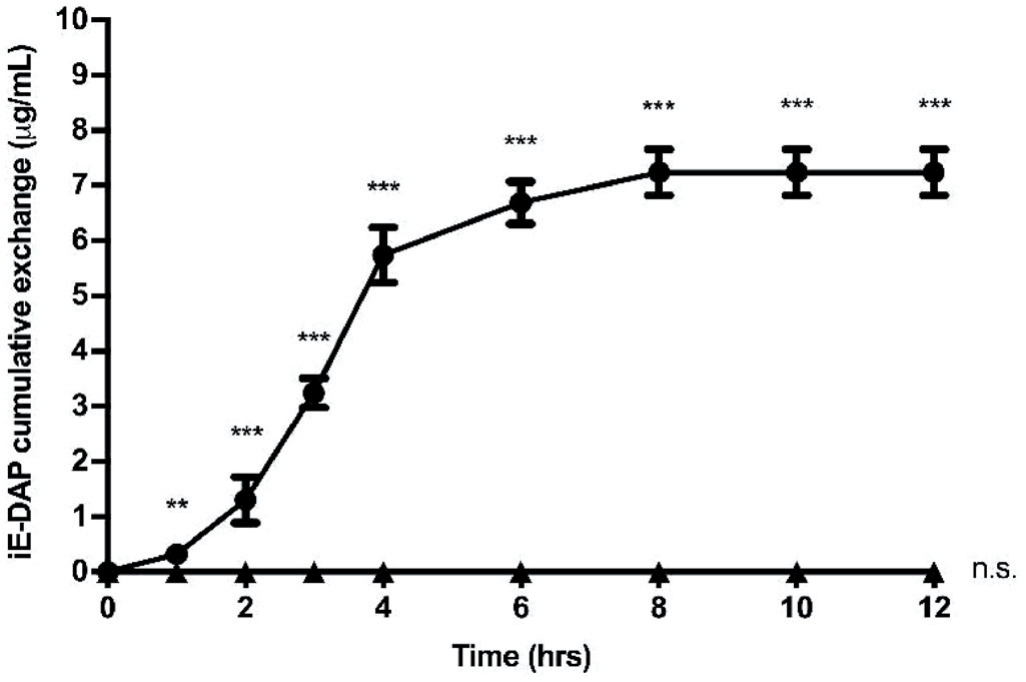
Cumulative exchange profile of iE-DAP agonist. 6 mg of PHBV-iE-DAP nanoparticles were incubated in 1 ml of PBS at 37 °C and with orbital shaking. At times 0, 1, 2, 3, 4, 6, 8, 10, and 12 h, it was centrifuged, and 50 µl aliquots were taken, which were quantified by UPLC (circles). As a negative control, empty NPs (triangles) were used. The results are expressed as the mean ± standard deviation of three independent experiments. One-way ANOVA was performed with the Bonferroni test as statistical analysis. **p < 0.01, ***p < 0.001.

The release profile of encapsulated iE-DAP agonist molecules from NPs was evaluated for 12 h in PBS at 37 °C. As shown in Figure 3, the agonist release profile behaves as a sigmoid curve, where more than 20% (1.4 µg) of the total amount of iE-DAP agonist is exchanged from the NPs during the first 2 h, and its maximum exchange occurs after 8 h. PHBV-NPs allow a constant release of iE-DAP, which would validate their use as drug delivery systems.

### 3.5. Cell internalization of PHVB NPs

As the immunostimulatory strategy proposed here consists of NPs encapsulating iE-DAP agonists, the cellular uptake of NPs by murine RAW 264.7 macrophages was evaluated. To this end, PHBV NPs loaded with Red Nile fluorophore were synthesized and used to visualize the amount of internalized NPs inside the cell. Then, the physicochemical properties of the synthesized PHBV-RN NPs were characterized, which did not change compared to empty NPs, as shown in Table 1. PHBV-RN NPs showed a negative surface charge of −6.5 mV and a size of 214.4 nm with a polydispersity value of 0.28, indicating a homogeneous size distribution similar to that previously reported (Peñaloza et al., 2017).

**Figure 4.**
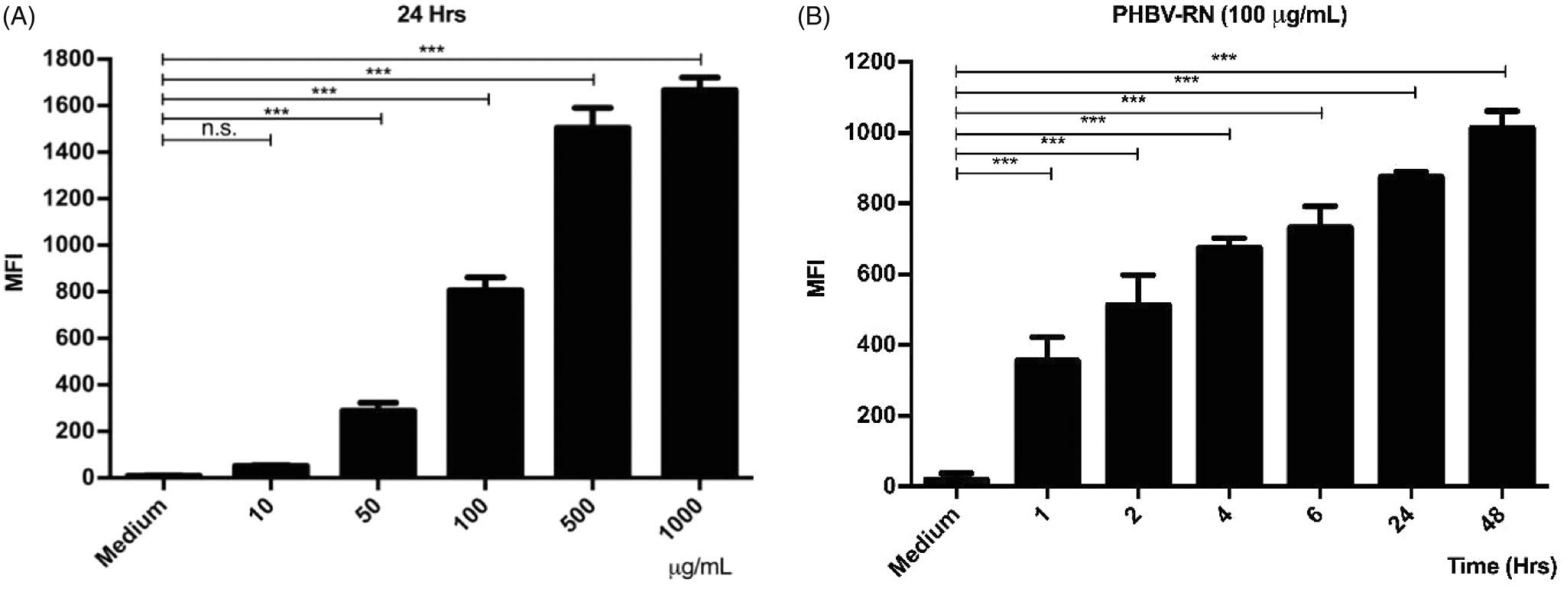
Internalization of PHBV-RN nanoparticles in RAW 264.7 cells. 5 × 10^5^ cells/well were seeded and incubated with different concentrations of PHBV-RN nanoparticles (10, 50, 100, 500, and 1000 µg/ml) in a fixed time of 24 h (A) and 100 µg of PHBV-RN at different times (1, 2, 4, 6, 24, and 48 h) (B) at 37 °C and 5% CO2. The culture medium was used as a negative control. The internalization of the nanoparticles was determined by flow cytometry (10,000 events). The mean fluorescence intensity (MFI) results are expressed as the average ± standard deviation of triplicate determinations of three independent experiments. One-way ANOVA was performed with the Bonferroni test as statistical analysis. *p < 0.05, **p < 0.01, ***p < 0.001.

Then, PHBV-RN NPs were added at different concentrations (10, 50, 100, 500, and 1000 µg/ml) and at different times (1, 2, 4, 6, 12, and 24 h) to the cells to be analyzed later by flow cytometry. Figure 4 shows that the internalization of the NPs is dependent on concentration and time. It is important to remark that results obtained by both experiments (concentration and time) showed congruence, where MFI (mean fluorescent intensity) were consistent when cells were incubated with a final concentration of 100 µg/ml PHBV-RN for 24 h.

### 3.6. Assessment of the potential cytotoxicity induced by NPs

**Figure 5.**
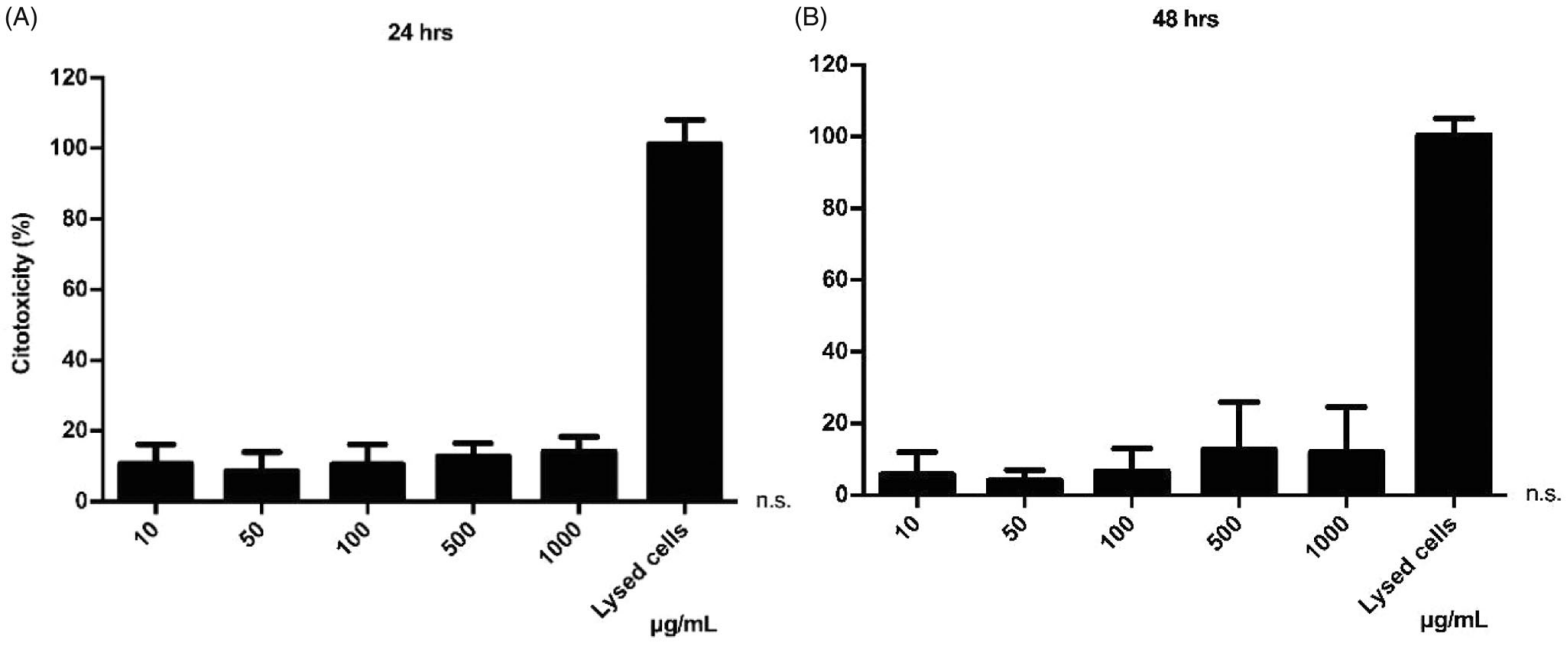
Cytotoxic effect of PHBV nanoparticles on RAW 264.7 cells. 5 × 10^5^ cells/well were seeded and treated with different concentrations of empty PHBV nanoparticles (10, 50, 100, 500, and 1000 µg/ml) for 24 h (A) and 48 h (B) at 37 °C and 5% CO_2_. The culture medium was used as a negative control, and the lysis buffer was added as a positive control. The LDH assay was used to determine cytotoxicity. The results are normalized concerning the control and expressed as the mean ± standard deviation of triplicate determinations of three independent experiments. One-way ANOVA was performed with the Bonferroni test as statistical analysis. n.s. = not significant.

The LDH assay was used to observe the potential cytotoxic effect of the iE-DAP-loaded NPs, as well as the concentration range in which they could be harmful to cells. In this assay, the cell membrane's integrity was evaluated, measuring the release of the LDH enzyme as an indirect indicator of cytotoxicity. The results indicate an average cytotoxicity level of 12.6% at 24 h and 8.1% at 48 h, respectively (Figure 5). This test showed that even at high concentrations of incubated NPs, no cytotoxic effect was shown (*p* > .05), suggesting that these NPs do not harmfully interact with the cell membrane, regardless of the concentration. Treatments were standardized compared to the control (baseline LDH exchange).

### 3.7. Cellular toxicity of NPs

**Figure 6.**
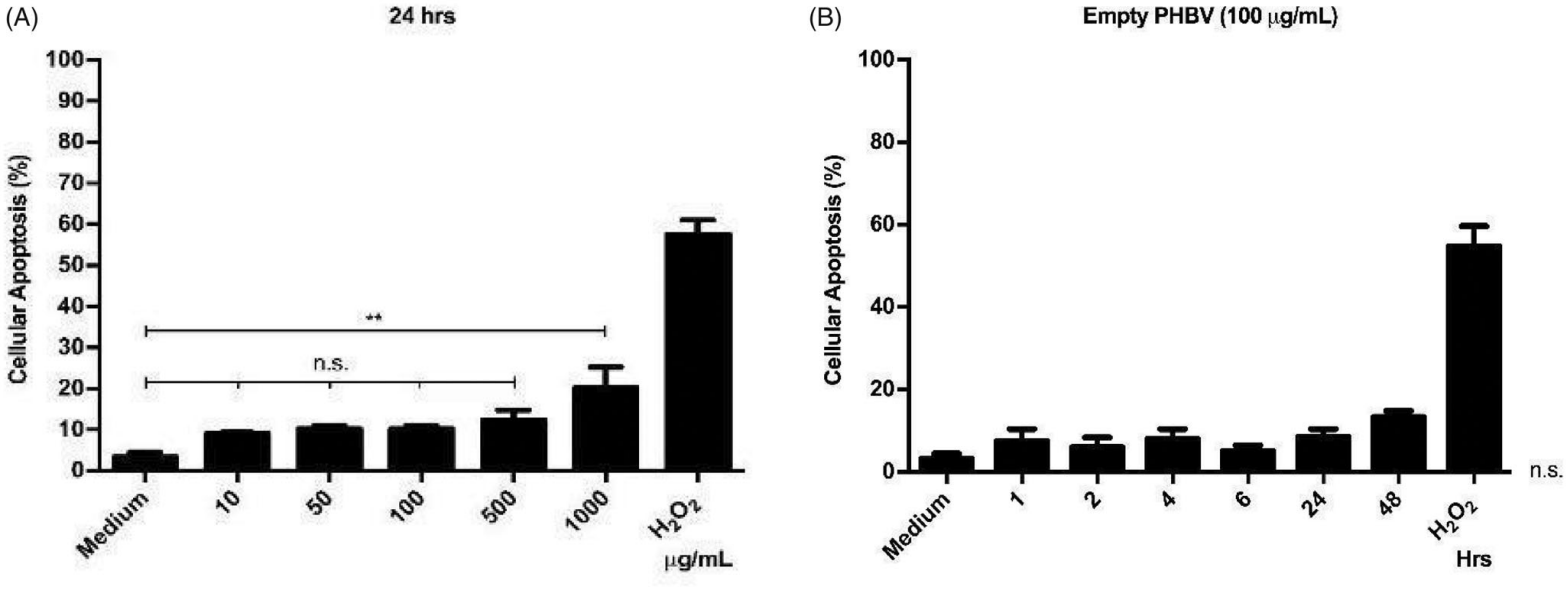
Cellular viability of RAW 264.7 cells in the presence of PHBV nanoparticles. 5 × 10^5^ cells/well were seeded and incubated with different concentrations of empty PHBV NPs (10, 50, 100, 500, and 1000 µg/ml) for 24 h (A) and different times (1, 2, 4, 6, 24, and 48 h) with 100 µg/ml of empty PHBV NPs (B). Cell viability was determined through the Annexin V assay by flow cytometry (10,000 events). Results are expressed as the mean ± standard deviation of triplicate determinations of three independent experiments. One-way ANOVA was performed with the Bonferroni test as statistical analysis. n.s. = not significant.

As different concentrations of PHBV NPs generate a discrete cytotoxic effect on RAW 264.7 cells. The next step was to determine whether these NPs could be triggering some apoptosis process. To this end, we evaluated the integrity of the cell membrane by annexin V, an apoptosis early marker. Annexin V preferentially binds to phospholipids that are translocated from the inside to the outside of the cell membrane in the early stages of apoptosis. Results obtained by flow cytometry indicate no significant changes in apoptosis levels concerning the negative control with only culture medium (except at a concentration of 1000 µg/ml) (Figure 6(A)). Furthermore, at a chosen concentration of 100 µg/ml, empty NPs do not show significant changes in apoptosis levels over time when measured at different times (1, 2, 4, 6, 24, and 48 h) (Figure 6(B)). As a positive control, a concentration of 1 µM H_2_O_2_ was used as an oxidizing agent capable of inducing apoptosis by over 50% in RAW 264.7 cells (Piao et al., 2011).

### 3.7. Evaluation of the activation of NF-κB factor by iE-DAP-loaded PHBV NPs

**Figure 7.**
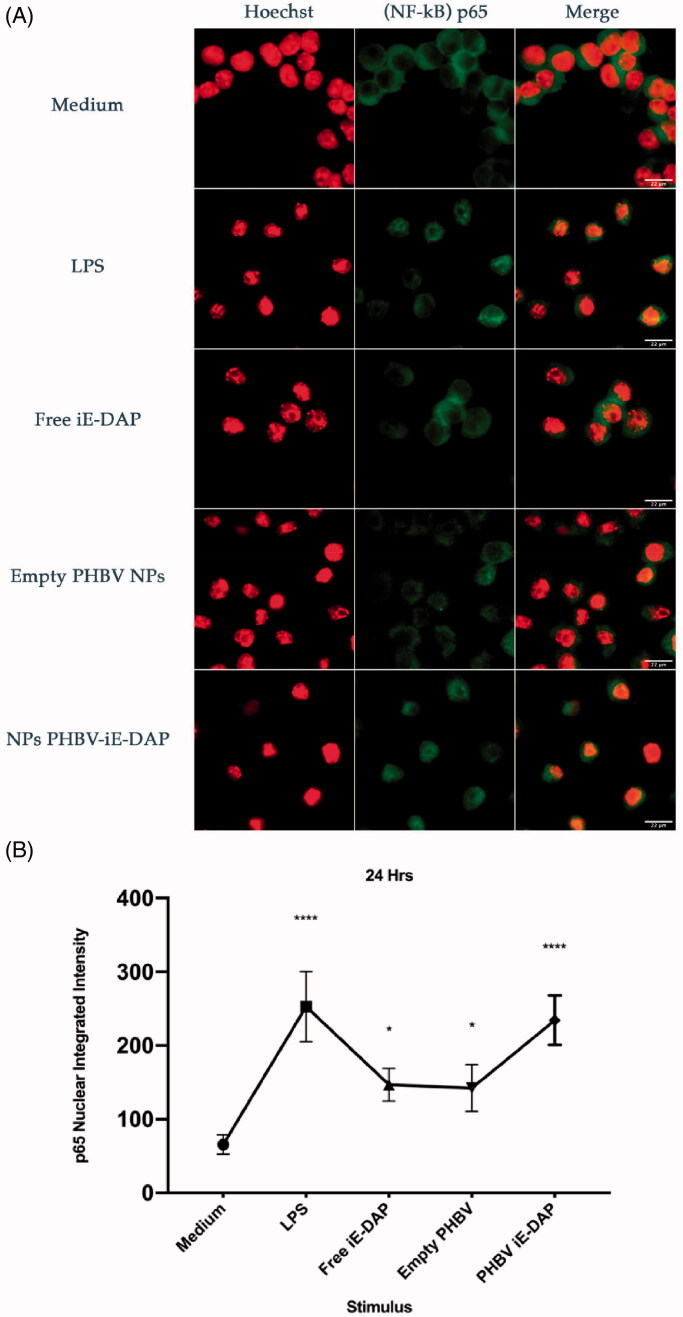
NF-κB translocation assay in RAW 264.7 cells. (A) Graphic representation of the nuclear translocation of p65 in immunofluorescence images (green). Culture medium (negative control), LPS 2.5 µg/ml (positive control), free iE-DAP ligand 7 µg/ml, empty PHBV NPs 100 µg/ml and NPB PHBV-iE-DAP 100 µg/ml for 24 h at 37 °C and 5% CO_2_. Representative cells were chosen by fluorescence microscopy, and their structures were labeled with Alexa-fluor 488 (p65 subunit, Green) and Hoechst 33342® (core, Blue changed to Red post image color, for better visualization). Each bar represents 22 µm. 100× magnification. Three experiments were performed independently. (B) The integrated intensity was quantified and analyzed by Fiji ImageJ. Results are expressed as the mean ± standard deviation of three separate field images. One-way ANOVA was performed with the Bonferroni test as statistical analysis. *p < .05, ****p < .0001.

NF-κB factor has been reported to be activated under LPS stimulation, causing its nuclear translocation and activating the transcription of pro-inflammatory genes (Baldwin, 1996). To verify whether iE-DAP-loaded NPs can activate the NF-κB factor, immunofluorescence assays were carried out by using an anti-p65 antibody to identify the p65 NF-κB component, which is regularly located in the cytosol of unstimulated cells (control). As shown in Figure 7(A), when 100 µg/ml of iE-DAP-loaded NPs for 24 h are incubated in RAW 264.7 cells (stable stock stored for 3–5 months at −20 °C, data not shown), it was possible to observe a significant increase in nuclear accumulation of p65 compared with an equivalent dose of the iE-DAP agonist in its soluble form. Empty NPs were also evaluated, showing a minimal accumulation in the nucleus (Figure 7(A,B)).

### 3.8. Evaluation of cytokines secretion elicited by iE-DAP-loaded PHBV NPs

**Figure 8.**
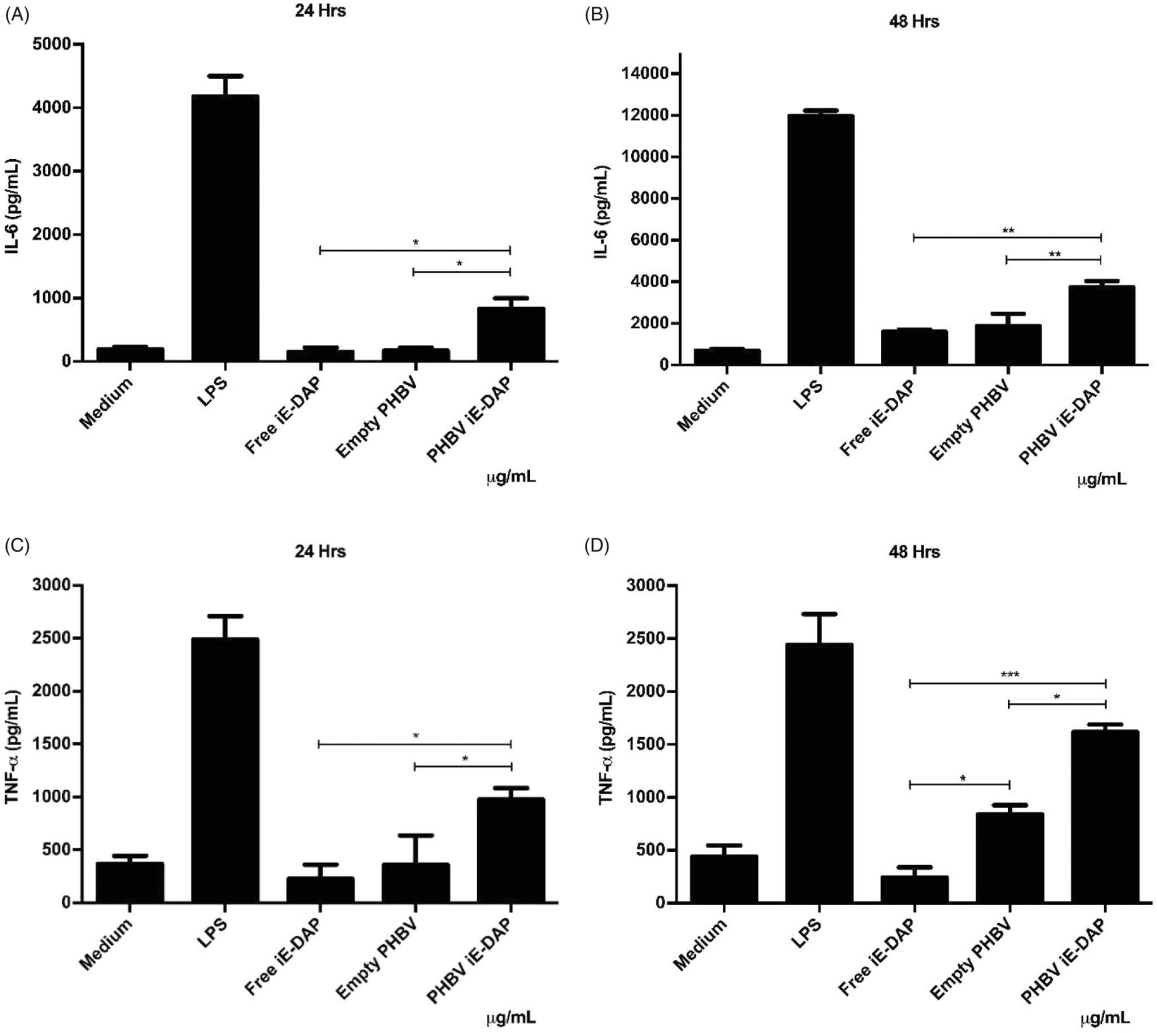
Quantification of pro-inflammatory cytokines in RAW 264.7 cells in vitro. 5 × 10^5^ cells/well were seeded and culture medium (control), LPS 2.5 µg/ml (positive control), free iE-DAP ligand 7 µg/ml, empty PHBV NPs 100 µg/ml, and PHBV-iE NPs were incubated iE-DAP 100 µg/mL for 24 h and 48 h at 37 °C and 5% CO_2_. Supernatants were taken, and concentrations of IL-6 at 24 and 48 h (A and B, respectively) and TNF-α at 24 and 48 h (C and D, respectively) were analyzed by a sandwich ELISA kit. Values are expressed as the mean ± standard deviation of triplicate determinations of three independent experiments. One-way ANOVA was performed with the Bonferroni test as statistical analysis. *p < .05, **p < .01, ***p < .001.

Finally, to explore the effect as a possible adjuvant, the secretion of two pro-inflammatory cytokines (IL-6 and TNF-α) was quantified by ELISA. Results in Figure 8 indicate that the iE-DAP agonist effect was improved by being encapsulated into PHBV NPs compared to its free form. Furthermore, it was possible to observe an increase of up to 5 times at 24 h and 2 times at 48 h of IL-6 levels, while TNF-α levels showed an increase of up to 4 times at 24 h and 16 times at 48 h compared to iE-DAP free form (*p* < .01).

## 4. Discussion

NPs have shown great potential for delivery system due to their multiple properties, such as the sustained supply of low molecular weight antigens toward a biological target for prolonged periods, to promote the protection and stabilization of the compound encapsulated by premature degradation (Hu et al., 2004; Vilos & Velasquez, 2012; Kumari et al., 2010).

In this work, we evaluate the use of PHBV NPs as a delivery system for NOD1 receptor activation. Here we showed that the intracellular fate of the NPs after endocytosis could favor the cytoplasmic delivery of the encapsulated agonist, increasing the activation efficiency of the NOD1 receptor, improving in this way its action compared to the soluble form of the agonist. PHBV polymers have recently started to be used similarly to well-known PLGA and PLA NPs, having the same physicochemical properties as the latter one and above all, due to its advantage of being an efficient alternative at low cost (Zhao et al., 2015).

As Table 1 illustrates, the nanoprecipitation process allows obtaining a high encapsulation performance of the agonist and reproducibility, having minimal differences in size, PdI, and Z potential between the synthesized batches. The high loading ability of the NPs to encapsulate iE-DAP peptides (70%) can be explained by the hydrophobic character of the agonists (partition coefficient octanol/water Log *P* = 1.22), which allows them to be encapsulated with high affinity by a hydrophobic nanoparticle such as PHBV (Zhao et al., 2015). In contrast, for hydrophilic compounds, such as the Ceftiofur antibiotic, PHBV has shown only 39.5% of encapsulation efficiency (Vilos et al., 2012).

Stable encapsulation of the molecules inside NPs is essential to use them in therapies and prevent the degradation of the encapsulated content. To this end, we measured the release of the agonist molecules in the PHBV NPs as a function of time (Figure 3). Although kinetics release in PHBV NPs has not been fully characterized, release mechanisms by other NPs has been reported (Xu et al., 2017). We could observe that our NPs behave as a triphasic type kinetic release profile, beginning with an erosion and rapid initial release of the agonist attached to the surface of the NPs, then the slow degradation of the polymer through the diffusion of water in the polymeric matrix and finally a sustained exchange over time (Soppimath et al., 2001; Hu et al., 2015). The peptide's full release occurs after 8 h under *in vitro* conditions; these values are comparable to those previously obtained by Babos et al. (2020) and Pavot et al. (2013). However, these values are contrasted by Silva et al. (2015), with different immunostimulant agents encapsulated in NPs, by presenting a slow cumulative release around 15 days. This behavior can be explained by the size and polymer concentration of the formulation, which influences the release rate of the compounds and degradation of the NPs (Tod et al., 2011).

Additionally, stability assessments of the NPs (Figure 2) indicate that size and surface charge did not vary over 4 weeks, which suggests that they contain an optimal size for a passive release of molecules as future use in *in vivo* applications (Peer et al., 2007). On the other hand, one of the advantages for biomedical use of these NPs is their negative surface charge granted by the carboxyl functional groups (Gupta et al., 1993). Those negatively charged groups avoid excessive protein binding and, therefore, prevent the stimulation of the innate immune system, resulting in a longer circulatory half-life. Besides, the negative charge minimizes non-specific binding with the cell surface and prevents aggregation itself (Nel et al., 2009).

There is a direct relationship between NPs physicochemical properties and an efficient capture by immune cells. While NPs among 500 nm in size are uptake by phagocytosis (Foster et al., 2001), it was reported that NPs with a size around 100–350 nm are internalized by clathrin-mediated endocytosis (Gratton et al., 2008; Peñaloza et al., 2017). Given that the size of PHBV NPs is around 198–215 nm (Table 1), it would probably be internalized through this process. After that, they might be found in intracellular compartments such as endosomes and lysosomes (Benfer & Kissel, 2012). These compartments are characterized by having a lower luminal pH of 6–6.5 in early endosomes and 4.5–5.2 in lysosomes (Yameen et al., 2014; Peñaloza et al., 2017). Our laboratory's previous results showed colocalization of these FITC-loaded NPs with late endosomes at 30 min and in lysosomes at 2 h, distinguishing the release of the FITC fluorophore in the human neutrophil cytoplasm of HL-60 and murine macrophages RAW 264.7 in 4 and 6 h, respectively (data not shown). The biological activity of iE-DAP-loaded NPs and the cytotoxicity were determined (Figure 5). Interestingly, after evaluating different concentrations and exposure times, NPs only generated about 12% of cytotoxicity, a percentage that is considered biocompatible for biological systems (Nel et al., 2009). However, tests with annexin V showed that at concentrations (>1 mg/ml) of NPs, the cells would have significant apoptosis levels in 20% (Figure 6(A)). This phenomenon could be caused by PHBV polymer remains after being degraded in endosomal compartments by acidic pH. One of its components, hydroxybutyric acid, is a ketone body that can be used as a favorable metabolic alternative substrate with protective effects against apoptosis mechanisms in PC12 cells (Cheng et al., 2010). Nevertheless, by contrast, the release of the alcohol groups of the PVA surfactant used in the NPs as a stabilizing agent could increase the intracellular oxidative stress and explain these apoptosis levels. For this reason, we decided to use a final concentration of 100 µg/ml that was non-cytotoxic at the concentration and time range evaluated (Figure 6(B)) (Ma et al., 2012; Silva et al., 2015).

In its free form, IE-DAP was able to induce the activation of NF-κB (Figure 7) and the consequent increase in pro-inflammatory cytokines (Figure 8), despite being impermeable to the membrane. This behavior could be due to cell entry mechanisms. A study in human epithelial cells HEK293 reported that a clathrin-mediated endocytosis process takes up NOD1 ligands such as iE-DAP and PHT1 (SLC15A4) in early endosomes favors their passage from the cytosol into cells (Lee et al., 2009). Another study on macrophages derived from mouse bone marrow also demonstrated the importance of PHT1 and showed that PEPT2 (SLC15A2) participates in the internalization process of NOD ligands (Hu et al., 2018).

Immunofluorescence results indicate that iE-DAP-loaded PHBV NPs induced NF-κB activation after translocation in the nucleus (Figure 7). This type of activation was observed to a lesser extent using the agonist in its free form and NPs empty than the control. NPs would improve peptide immunostimulatory properties, probably due to reduced degradation of the peptide in the culture medium or enhanced cell entry of the PHBV-iE-DAP NPs, transporting its content more efficiently to NOD1 intracellular receptors. Furthermore, we showed here that the secreted amounts of IL-6 and TNF-α pro-inflammatory cytokines are higher by iE DAP-loaded NPs than the peptide in the free form (Figure 8). Those values are consistent with the work already reported (Wischke et al., 2012; Pavot et al., 2013). Nevertheless, the secreted levels of TNF-α by empty PHBV NPs at 48 h are significant, indicating that they could also be acting as an inducer. Results of Pavot et al. (2013, 2014) exposing empty PLA NPs in human MoDCs did not produce a significant secretion of TNF-α. They showed that the degradation of PVA would be contributing to the formation of oxidative stress, activation of apoptosis and secretion of TNF-α pro-inflammatory cytokine (Cheng et al., 2015).

On the other hand, our laboratory results suggested phagocytosis as the PHBV NPs internalization mechanism in immune cells. To date, a specific receptor, or a possible mechanism of activation of the cytokine TNF-α through PHBV NPs that reflects this secretion is still unknown.

Despite this evidence, this pleiotropic cytokine can act both autocrine and paracrine way through TNFR2 membrane receptors in immune cells, explaining its effect in the activation and release levels observed at 48 h (Figure 8(D)) (Gane et al., 2016).

Taken together, these behaviors could lead to a constant overproduction of pro-inflammatory cytokines, generating a hyperinflammatory state at the systemic level, causing damage and dysfunction of tissues and organs (Cartwright et al., 2007). This result suggests that there must be a delicate balance between the innate immune response, which must be enough to eliminate the pathogen and the negative feedback system to prevent pathological inflammation.

Therefore, subsequent investigations could determine the precise range of peptide release from NPs as a function of time. This profile can be modified using PHBV polymers with low molecular weight or PHBV with a higher valerate composition to retain compounds with higher affinity by hydrophobic compounds (Göpferich, 1996). Moreover, the saturation point of the peptide loading of NPs should also be determined to increase or decrease its encapsulation and maintain adequate concentration levels over time for therapeutic purposes. Some studies have shown that NOD1 agonists can be used prophylactically as a complement of antibiotics, which substantially improves survival in microbial sepsis models (Mine et al., 1983).

## 5. Conclusions

In the present work, we proposed a new formulation composed of a NOD1 agonist encapsulated into biocompatible PHBV NPs that might control the immune response at cellular level. Our experimental results showed that PHBV NPs could encapsulate iE-DAP peptide without altering its immunogenic properties. Moreover, we demonstrate that the iE-DAP agonist effect was improved by encapsulation compared to its soluble form, by promoting an activation of NOD1 receptor in macrophages.
